# Peripheral neurotoxicity induced by albumin-bound paclitaxel: a case report

**DOI:** 10.3389/fonc.2024.1440205

**Published:** 2024-09-04

**Authors:** Xiaojing Li, Liuting Wu, Dunyao Bai, Juan Li

**Affiliations:** ^1^ Department of Pharmacy, Puren Hospital of Wuhan University of Science and Technology, Wuhan, Hubei, China; ^2^ Department of Pharmacy, Hubei Cancer Hospital, Tongji Medical College, Huazhong University of Science and Technology, Wuhan, Hubei, China

**Keywords:** albumin-bound paclitaxel, case report, liposomal paclitaxel, non-small cell lung cancer, peripheral neurotoxicity

## Abstract

**Background:**

Despite its excellent therapeutic efficacy, albumin-bound paclitaxel often leads to peripheral neurotoxicity, significantly affecting patients’ quality of life. This study reported a case of non-small cell lung cancer (NSCLC) with peripheral neurotoxicity induced by albumin-bound paclitaxel.

**Case presentation:**

A 70-year-old male was admitted to the Hubei Cancer Hospital complaining of left-side limb weakness and numbness for one month following the first cycle of albumin paclitaxel plus cisplatin plus tislelizumab regimen for the right-side NSCLC in December 2021. Chest CT displayed a soft tissue density mass in the apical segment of the right upper lung lobe (5.5×4.9 cm^2^). Immunohistochemistry results and CT-guided percutaneous lung biopsy confirmed NSCLC stage cT_3_N_X_M_X_. The pain and numbness in both feet were alleviated after the first cycle of this regimen of liposomal paclitaxel 240 mg plus cisplatin 90 mg plus tislelizumab 200 mg. After four treatment cycles, the tumor treatment was evaluated as partial response (PR), and the tumor lesion became 2.9×2.7 cm^2^.

**Conclusion:**

The regimen containing liposomal paclitaxel, cisplatin, and tislelizumab alleviated the symptoms of peripheral neurotoxicity induced by albumin-bound paclitaxel in an NSCLC case, which may be a potential therapeutic option.

## Introduction

Albumin-bound paclitaxel is frequently used in various cancer treatments, including in non-small cell lung cancer (NSCLC), due to its strong targeting capability and low toxicity (1). With its frequent use, its adverse reactions have received attention. Peripheral neuropathy is the most frequent adverse event induced by paclitaxel and requires drug modification or discontinuation (2, 3). Symptoms of peripheral neuropathy depend on the types of neurons (sensory, motor, or autonomic neurons) affected. The mechanism of neuronal injury may be due to altered microtubule dynamics, mitochondrial malfunction, and inflammation of peripheral neurons (4). As peripheral neurotoxicity is one of the most common adverse reactions associated with paclitaxel therapy, finding effective solutions is crucial for enhancing patients’ treatment experiences. Additionally, effectively managing peripheral neurotoxicity can reduce the need for dose reductions or treatment interruptions due to toxicity, thereby helping to maintain or improve treatment efficacy. Thus, identifying and implementing solutions to Albumin-bound paclitaxel-induced peripheral neurotoxicity is essential for enhancing patients’ quality of life, improving treatment sustainability, and promoting favorable clinical outcomes. However, the current research on this topic remains very limited. Therefore, this study reported a case of peripheral neurotoxicity caused by albumin-bound paclitaxel in an NSCLC patient.

## Case description

A 70-year-old male was admitted to the Hubei Cancer Hospital with complaints of left-sided limb weakness and numbness persisting for one month after completing the first cycle of albumin paclitaxel, cisplatin, and tislelizumab treatment for right-sided NSCLC in December 2021. Vital signs were stable upon admission, with a temperature of 36.5°C, pulse rate of 85 beats/min, respiratory rate of 19 breaths/min, and blood pressure of 135 mmHg/85 mmHg. He had a medical history of hypertension and cerebral infarction. Blood tests revealed platelet count of 212×10^9^/L (normal range: 150-450×10^9^/L), hemoglobin level of 105 g/L (normal range: 13-18 g/L), white blood cell count of 3.3×10^9^/L (normal range: 4500-11000/L), and red blood cell count of 3.64×10^12^/L (normal range: 4.7-6.1×10^6^/L). Chest CT revealed a soft tissue density mass in the apical segment of the right upper lung lobe (5.5×4.9 cm^2^). CT-guided percutaneous lung biopsy was performed on December 1, and the pathological diagnosis was non-small cell carcinoma (lung) (stage cT3NXMX), which was consistent with squamous cell carcinoma combined with immunohistochemistry. The immunohistochemical findings were CD56 (-), CK5/6 (+), CK7 (-), Ki-67 (+, about 30%), NapsinA (-), P40 (+), P63 (+), and TTF-1 (-).

**Figure 1:**
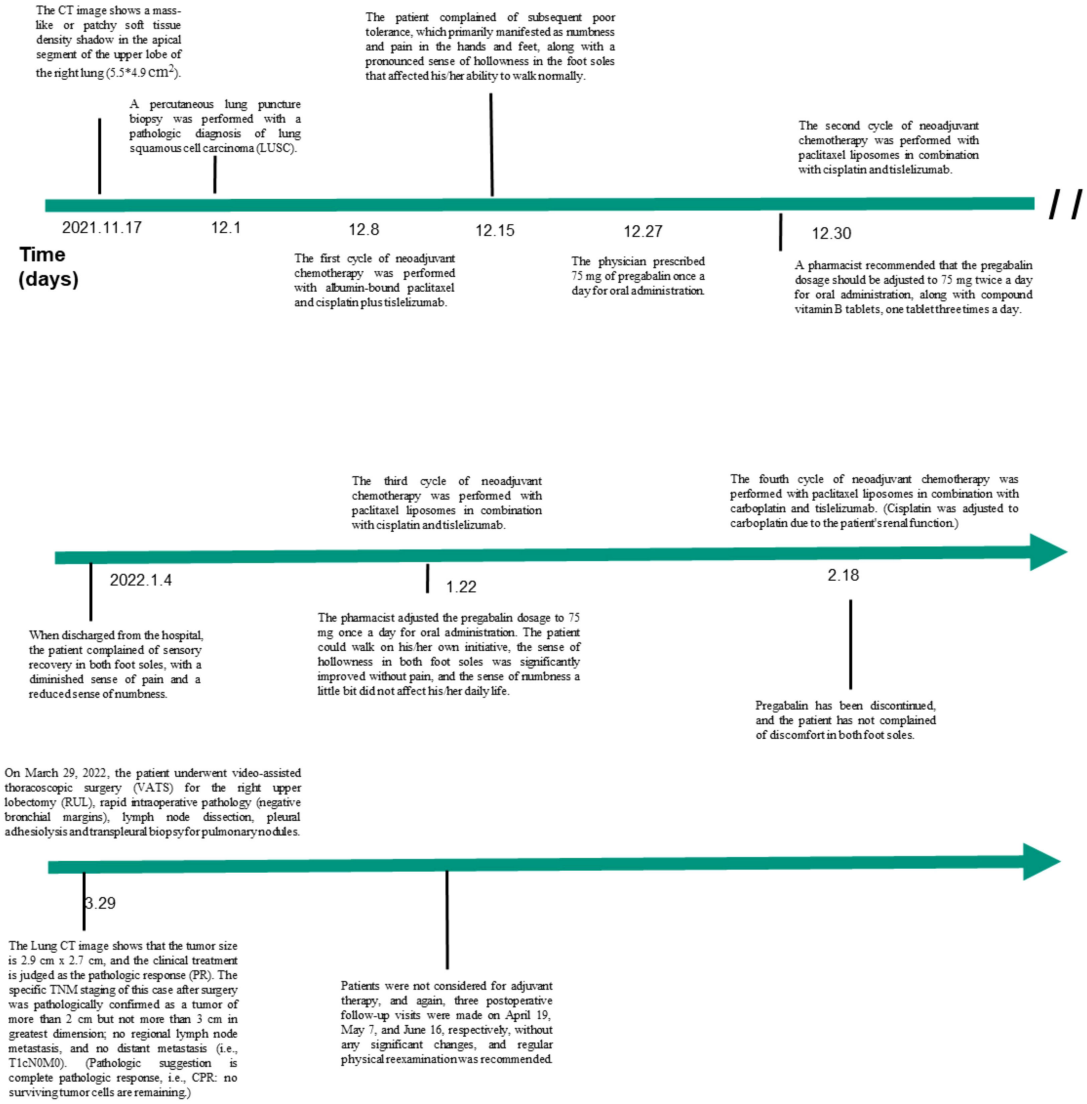
Timeline.

The PET-CT examination showed a soft tissue mass in the upper lobe of the right lung with increased metabolism, as well as several regional lesions. Preoperative chemotherapy and immunotherapy were recommended. Surgery was determined according to the post-treatment evaluation results. The patient and his family agreed to central vein catheterization on December 7, and albumin-bound paclitaxel with cisplatin and tislelizumab were given for one cycle on December 8, in addition, he had been taking Centrum on his own. The patient reported poor tolerance, mainly with hand and foot numbness and pain and bone marrow suppression. Then, he was given pregabalin (75 mg) to alleviate numbness and pain in the hands and feet. However, symptom relief was not significant. Despite the patient’s refusal of further albumin-bound paclitaxel, liposomal paclitaxel (240 mg), cisplatin (90 mg), and tislelizumab (200 mg) were administered following a multidisciplinary team (MDT) discussion and literature review. Following two cycles, ambulation improved significantly, and pain and numbness reduced. After four cycles, partial response was observed, with tumor size reduced to 2.9×2.7 cm^2^. Detailed diagnosis and treatment procedures are shown in **Figure 1**.

## Discussion

This study presented a case of peripheral neuropathy induced by albumin-bound paclitaxel in conjunction with cisplatin and tislelizumab in an NSCLC patient. Upon replacing albumin-bound paclitaxel with liposomal paclitaxel in the treatment regimen, the patient experienced gradual alleviation of neuropathic symptoms, achieving partial relief after four treatment cycles.

The severity of paclitaxel-induced peripheral neuropathy varies depending on multiple factors, including age, cumulative drug exposure, statin use, and low hemoglobin levels (5). In this case, advanced age and mild anemia (grade I) likely contributed to the neuropathic symptoms. According to the National Cancer Institute-Common Toxicity Criteria (NCI-CTC) and the World Health Organization (WHO) grading criteria, the peripheral neuropathy induced by albumin-bound paclitaxel is categorized as grade 3. These peripheral neurological symptoms can be managed by dose reduction or discontinuation of paclitaxel.

A lower limit dose of 135 mg/m^2^ paclitaxel liposome was given to this patient, which resulted in a good treatment effect and patient tolerability. Paclitaxel liposome is prepared by encapsulating paclitaxel in a spherical-shaped nano-sized lipid vesicle. Paclitaxel liposome reduced the toxicity, possibly due to its low distribution in the central nervous system and peripheral neurons (6). Paclitaxel liposomes mainly accumulate in the reticuloendothelial system in the liver, spleen, lungs, and lymphatic tissues, improving its therapeutic index and reducing its toxicity (7). In patients who are highly tolerable to paclitaxel liposome, it would be a better choice. Patients need to be educated to report promptly if any numbness or tingling in the hands and feet during treatment arises to prevent the worsening of neuropathy. Patients should also be educated to prevent falls, bumps, burns, frostbite, and sharp injuries. Patients should avoid touching cold objects, wear gloves and thick socks when going out, and reduce direct contact with iron items.

Although allergy and other adverse reactions to albumin-bound paclitaxel are far less common than with paclitaxel, the occurrence of peripheral neurotoxicity is uncommon but never as serious as in this patient, who could no longer walk. Cisplatin induces some neurotoxicity, but it is usually not acute and occurs after a few cycles. On the other hand, acute neurotoxicity has been reported with paclitaxel (3, 4, 6). The patient himself felt some discomfort during albumin-bound paclitaxel infusion and waited until the discomfort increased a little but could tolerate it without complaining, and became more and more uncomfortable after going home, finally affecting walking. The neuropathy disappeared after switching to liposomal paclitaxel, suggesting that the cisplatin-induced neuropathy had not yet taken place.

Although the mechanism of peripheral neuropathy caused by paclitaxel is not fully understood, some mechanisms have been proposed. Firstly, paclitaxel may directly damage neurons, affecting the transmission of electrical impulses through neuronal axons and resulting in functional disturbances (8). Secondly, paclitaxel disrupts the microtubule structure of the cell cytoskeleton, inhibiting the depolymerization of microtubule proteins and interfering with tumor cell mitosis (9). In addition, the production of free radicals, ion channel abnormalities, and activation of astrocytes were also related to the neuropathic effects of paclitaxel (10).

The strength of the case reported here was happened in China and the prompt identification of albumin-bound paclitaxel as being the possible cause of numbness and pain in the hands and feet and the switch to paclitaxel liposome, allowing the completion of the planned anticancer treatment while avoiding the toxicity albumin-bound paclitaxel. Still, limitations should also be considered, including only one patient being included and a lack of explanation for the peripheral neurotoxicity being observed. After the first cycle of treatment, the patient complained that he could not stand and walk and was admitted with crutches. He obviously felt hollow and numb pain like a dry turtle shell on the sole of his feet. The patient also had a strong aversion to using albumin-paclitaxel again, and he refused further treatment. Although ceasing treatments would probably lead to a poor prognosis, patient-reported outcomes and wishes must be taken into consideration. Finally, no specific questionnaire was used. The diagnosis was based on the Chief physician’s clinical experience.

In conclusion, this study reported a case of peripheral neuropathy induced by albumin-bound paclitaxel in the treatment of NSCLC. The implementation of liposomal paclitaxel, in conjunction with cisplatin and tislelizumab, demonstrated promising outcomes in alleviating neuropathic symptoms.

## Data Availability

The original contributions presented in the study are included in the article/supplementary material. Further inquiries can be directed to the corresponding author.
